# MicroRNA-204 Deficiency in Human Aortic Valves Elevates Valvular Osteogenic Activity

**DOI:** 10.3390/ijms21010076

**Published:** 2019-12-20

**Authors:** Rui Song, Yufeng Zhai, Lihua Ao, David A. Fullerton, Xianzhong Meng

**Affiliations:** Department of Surgery, University of Colorado Denver, Aurora, CO 80045, USA; rui.song@ucdenver.edu (R.S.); yufeng.zhai@cuanschutz.edu (Y.Z.); lihua.ao@cuanschutz.edu (L.A.); david.fullerton@cuanschutz.edu (D.A.F.)

**Keywords:** aortic valve calcification, MicroRNA, TGF-β1, alkaline phosphatase, gene knockdown

## Abstract

Aortic valve interstitial cells (AVICs) play a major role in valvular calcification associated with calcific aortic valve disease (CAVD). Although AVICs from diseased valves display a pro-osteogenic phenotype, the underlying mechanism causing this remains unclear. MicroRNA-204 (miR-204) is a negative regulator of osteoblast differentiation. We sought to analyze miR-204 expression in diseased human aortic valves and determine the role of this miR in AVIC osteogenic activity associated with CAVD pathobiology. In situ hybridization and PCR analysis revealed miR-204 deficiency in diseased valves and in AVICs from diseased valves. MiR-204 mimic suppressed alkaline phosphatase (ALP) expression and calcium deposition in AVICs from diseased valves. MiR-204 antagomir enhanced ALP expression in AVICs from normal valves through induction of Runx2 and Osx, and expression of miR-204 antagomir in mouse aortic valves promoted calcium deposition through up-regulation of Runx2 and Osx. Further, miR-204 mimic suppressed the osteogenic responses to TGF-β1 in AVICs of normal valves. In conclusion, miR-204 deficiency contributes to the mechanism underlying elevated osteogenic activity in diseased aortic valves, and miR-204 is capable of reversing the pro-osteogenic phenotype of AVICs of diseased valves and suppressing AVIC osteogenic response to stimulation. Exogenous miR-204 may have therapeutic potential for inhibiting valvular calcification associated with CAVD progression.

## 1. Introduction

Calcific aortic valve disease (CAVD) is one of the most prevalent cardiovascular diseases in elderly people (65 or older). In many patients this disease progresses to symptomatic severe aortic stenosis, a clinical entity for which costly aortic valve replacement is needed. The pathobiology of CAVD involves nodular calcification of valvular leaflets, and progressive valvular calcification is a major cause of morbidity and mortality in patients with CAVD [1,2]. However, clinically significant aortic stenosis takes years to develop. The slow progressive course of CAVD affords a wide window for pharmacological intervention to halt disease progression. Unfortunately, gaps in our knowledge of the mechanisms underlying CAVD progression impede the development of pharmacological therapies [3,4].

Aortic valve interstitial cells (AVICs) are the predominant cells in aortic valvular leaflets. In normal human aortic valves, AVICs are mostly quiescent fibroblasts that play an important role in maintaining valvular homeostasis. In the pathological condition of CAVD, however, AVICs are key cells involved in valvular calcification [5,6,7,8]. AVICs are capable of synthesizing and secreting matrix proteins, cytokines, and growth factors [9,10]. It has been documented that activated AVICs and AVICs of diseased aortic valves express osteoblast biomarkers including alkaline phosphatase (ALP), osteopontin, bone sialoprotein, osteocalcin, and osteoblast-specific transcription factors [6,11,12,13,14]. Interestingly, AVICs from diseased human aortic valves display a pro-osteogenic phenotype with enhanced expression of pro-osteogenic factors, such as TGF-β1 and BMP-2, and osteogenic biomarkers ALP and Runt-related transcription factor 2 (Runx2) in the baseline and in response to stimulation. The current scientific consensus is that valvular calcification involves acquisition of osteogenic activity by AVICs to become “osteoblast-like” cells [15]. An important question that has to be addressed is what causes AVICs to change into a pro-osteogenic phenotype.

Transforming growth factor-beta 1 (TGF-β1) and bone morphogenetic protein-2 (BMP-2) are identified as pro-osteogenic factors that mediate the osteogenic activity in vascular cells [16,17,18]. Previous studies found that calcified aortic valves have higher levels of TGF-β1 in comparison to non-calcified aortic valves [19]. TGF-β1 has been shown to up-regulate ALP expression and osteogenic activity in human AVICs [20,21,22]. Thus, TGF-β1 appears to play a mechanistic role in promoting valvular calcification. Understanding of the molecular mechanism by which TGF-β1 up-regulates the osteogenic activity in human AVICs will provide insights into the mechanism underlying the progression of aortic valve calcification.

Runx2 and Osterix (Osx) mediate osteoblast differentiation [23,24]. The significance of Runx2 and Osx in aortic valve calcification is highlighted by the observation that the expression of these two osteogenic transcription factors in human AVICs is up-regulated by pro-osteogenic stimuli [6,21]. While ALP plays a critical role in valvular calcification, the link of Runx2 and Osx to the up-regulation of ALP expression and osteogenic activity in the aortic valve remains to be determined.

MicroRNAs (miRs), a family of small (21–23 nucleotide) and non-coding RNAs, play a mechanistic role in the pathobiology of cardiovascular diseases and are implicated as potential therapeutic targets [25]. A number of studies identified altered miR expression in calcified aortic valves [26,27,28]. In addition, several miRs were found to modulate AVIC osteogenic activity or osteogenic responses to pro-osteogenic stimulation. In this regard, miR-30b is reported to attenuate BMP-2-induced ALP expression and osteogenic activity [26], and miR-204 is capable of down-regulating BMP-2-induced “osteoblast-like” differentiation [29]. Further, inhibition of miR-34a suppresses aortic valve calcification caused by wire injury in mice [30]. With regard to miR-204, this miR has been shown to down-regulate Runx2 expression associated with mesenchymal progenitor cell differentiation to osteoblast, indicating that this miR may function as a negative regulator of osteoblast differentiation [31]. Our recent analysis of miR profile in human AVICs demonstrates that AVICs from human aortic valves affected by CAVD have higher levels of miR-486 and lower levels of miR-204 and that pro-osteogenic factors, such as TGF-β1 and BMP-2, down-regulate miR-204 expression in AVICs from normal human aortic valves [32]. It is likely that miR-204 down-regulation plays a mechanistic role in promoting valvular osteogenic activity. Currently, it is unclear whether the expression of miR-204 is altered in diseased human aortic valves and whether modulation miR-204 level suppresses the osteogenic activity in AVICs of diseased human aortic valves.

Using in vitro and ex vivo models, we sought to test the hypothesis that miR-204 deficiency in diseased aortic valves exacerbates valvular calcification. The purpose of this study was to determine: (1) whether miR-204 level is reduced in human aortic valves affected by CAVD, (2) the effect of miR-204 on the osteogenic activity of AVICs from diseased valves, and (3) the mechanism by which miR-204 modulates valvular calcification.

## 2. Results

### 2.1. Diseased Human Aortic Valves are Deficient in MiR-204

**Figure 1:**
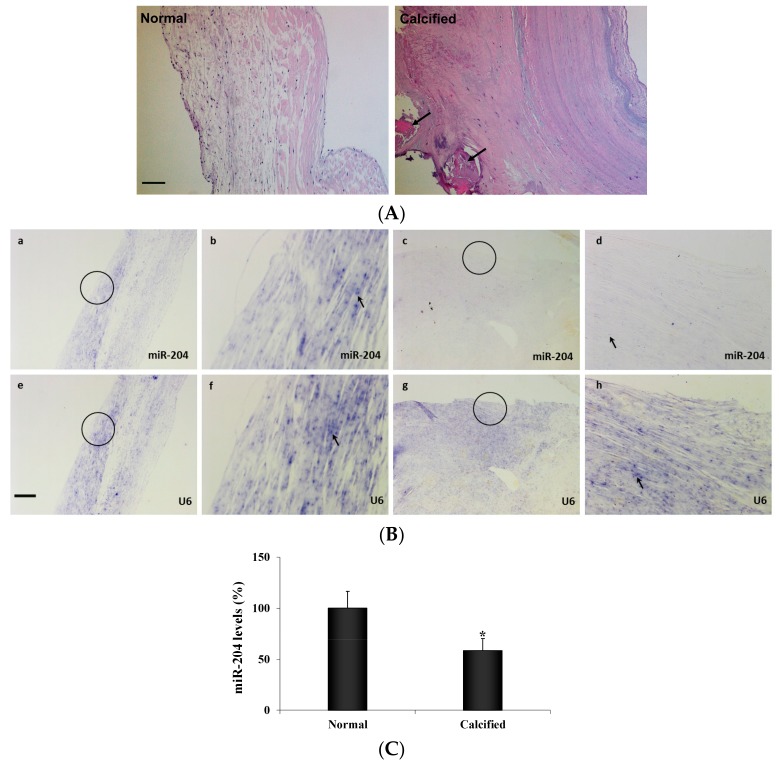
MiR-204 levels are lower in diseased human aortic valves. (**A**) Hematoxylin and eosin stain images (10× objective) of normal and calcified human aortic valve leaflets show that calcified aortic valve leaflets loss tri-layered morphology. Calcification nodules (arrows) are present in moderately calcified aortic valve leaflets. Scale bar = 100 µm. (**B**) Normal and calcified human aortic valve tissue sections were incubated with a full length DIG-labeled LNA probe to miR-204 (a–d) and a DIG-labeled LNA probe specific for the non-coding small nuclear RNA U6 as internal control (e–h). MiR-204 (blue, arrow) is markedly lower in diseased valves (c and d) compared with normal valves (a and b). Original magnification is 4x objective in a, c, e and g, and 20× objective in b, d, f and h. Scale bar = 100 µm. (**C**) Aortic valve interstitial cells (AVICs) isolated from normal and calcified human aortic valves were analyzed for miR-204 levels by real-time PCR. Quantitative mRNA data confirmed that miR-204 levels are lower in AVICs from calcified valves. Mean ± SE; *n* = 8 distinct cell isolates; * *p* < 0.05 vs. AVICs of normal valves.

Calcification nodules were observed in moderately calcified aortic valve leaflets from patients with CAVD (Figure 1A). In situ hybridization demonstrated the presence of miR-204 in human aortic valves. However, significantly lower levels of miR-204 were observed in diseased aortic valves (Figure 1B). Expression of miR-204 in human AVICs was confirmed using Real-time qRT-PCR (Figure 1C). Similarly, AVICs from diseased aortic valves had markedly lower levels of miR-204 (−40.8%, Figure 1C) in comparison to AVICs from normal aortic valves.

### 2.2. MiR-204 Mimic Suppresses the Osteogenic Activity in AVICs from Diseased Valves

**Figure 2:**
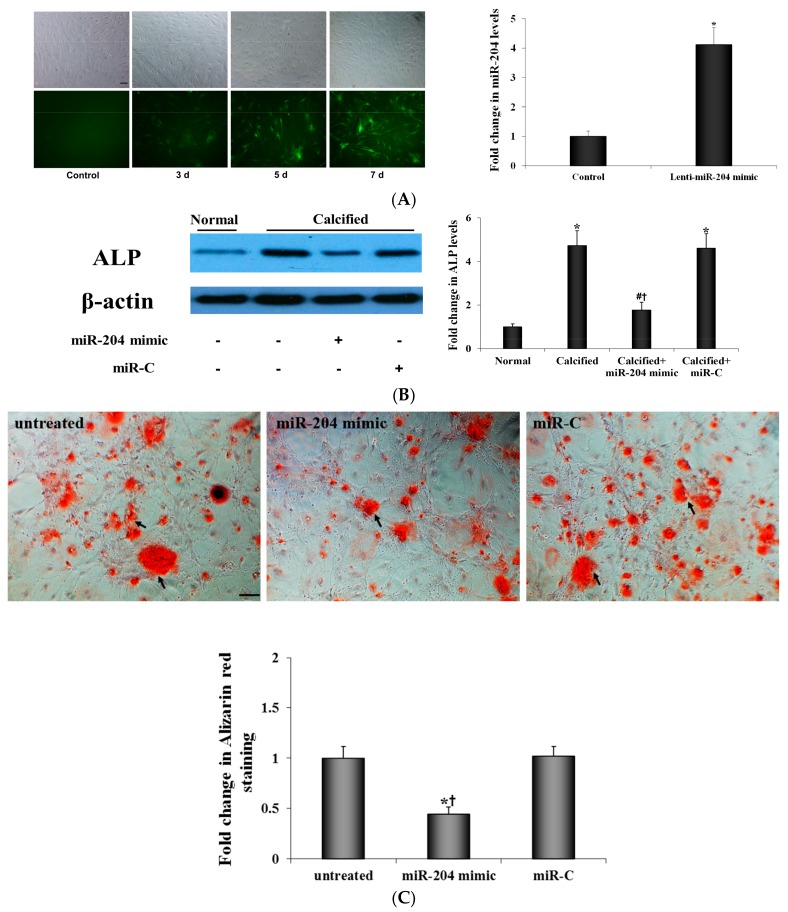
MiR-204 mimic suppresses the osteogenic activity in AVICs from diseased valves. (**A**) Human AVICs were infected with lentivirus expressing miR-204 mimic. Representative images show lentiviral infection efficiency (GFP expression) over time in human AVICs. Data of real-time qRT-PCR analysis show markedly elevated miR-204 levels in cultures treated with lentiviral miR-204. Scale bar = 200 μm. Mean ± SE; *n* = 6 distinct cell isolates; * *p* < 0.05 vs. Control (culture infected by lentiviral miR-C). (**B**) AVICs from diseased human aortic valves were treated with miR-204 mimic (5 nM) or irrelevant oligonucleotide (miR-C, 5 nM) for 72 h. Representative immunoblots of 6 separated experiments using distinct cell isolates and densitometric data show that miR-204 mimic suppressed the expression of ALP in AVICs of diseased valves. Mean ± SE; * *p* < 0.05 vs. normal AVICs; # *p* < 0.05 vs. untreated calcified valve AVICs; ^†^ *p* < 0.05 vs. calcified valve AVICs+miR-C. (**C**) AVICs from diseased human aortic valves were untreated or infected with lentivirus that expresses miR-204 mimic or miR-C for seven days, and then cells are incubated in a conditioning medium (growth medium supplemented with 10 mmol/L β-glycerophosphate, 10 nmol/L vitamin D3, 10 nmol/L dexamethasone and 8 mmol/L CaCl_2_) for 14 days. Expression of miR-204 mimic suppressed calcium deposit formation. Scale bar = 200 μm. Mean ± SE; *n* = 6 distinct cell isolates; * *p* < 0.05 vs. untreated AVICs; ^†^ *p* < 0.05 vs. AVICs treated with miR-C.

To determine whether correction of miR-204 levels has an effect on the osteogenic activity of AVICs from calcified aortic valves, we infected AVICs from diseased valves with lentivirus that expresses miR-204 mimic and examined ALP expression and calcium deposit formation. As shown in Figure 2A, lentiviral expression of miR-204 mimic increased miR-204 levels in human AVICs from diseased valves. The result in Figure 2B shows that miR-204 mimic markedly reduced ALP protein levels in AVICs from diseased valves. More importantly, expression of miR-204 mimic in AVICs from diseased valves suppressed their formation of calcium deposits, while expression of irrelevant miR had no effect (Figure 2C). Thus, reduced miR-204 levels appear to be responsible for the elevated osteogenic activity of AVICs from diseased valves, and correction of miR-204 deficiency is capable of reversing, at least partly, the pro-osteogenic phenotype of AVICs of diseased valves.

### 2.3. MiR-204 Antagomir Enhances the Expression of Osteogenic Biomarkers in AVICs from Normal Human Aortic Valves

**Figure 3:**
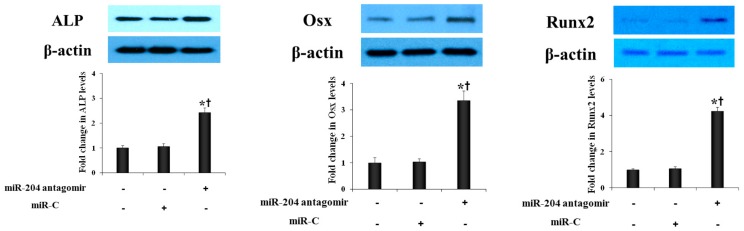
MiR-204 antagomir enhances the expression of osteogenic biomarkers in AVICs from normal aortic valves. AVICs from normal human aortic valves were treated with miR-204 antagomir (50 nM) or irrelevant oligonucleotide (miR-C, 50 nM) for 72 h. Representative immunoblots and densitometric data show that miR-204 antagomir up-regulates the levels of Runx2, Osx and ALP. Mean ± SE; *n* = 6 distinct cell isolates; * *p* < 0.05 vs. untreated control; ^†^ *p* < 0.05 vs. cells treated with miR-C.

To further determine the role of miR-204 in modulation of AVIC osteogenic activity, we examined the effect of miR-204 antagomir on the expression of osteogenic biomarkers in AVICs from normal human aortic valves. As shown in Figure 3, transfection of cells with miR-204 antagomir increased the protein levels of Runx2, Osx and ALP. Thus, transient down-regulation of miR-204 increases the osteogenic activity in human AVICs.

### 2.4. MiR-204 Up-Regulates the Expression of Runx2 and Osx in Human AVICs to Induce the Transition to Pro-Osteogenic Phenotype

**Figure 4:**
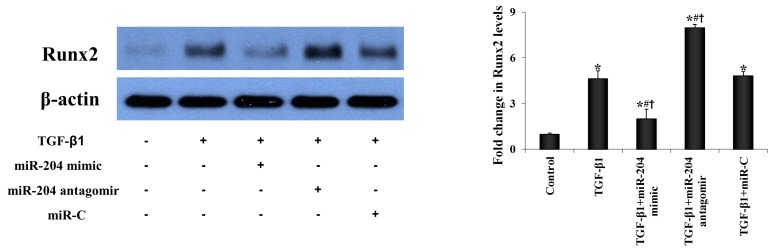

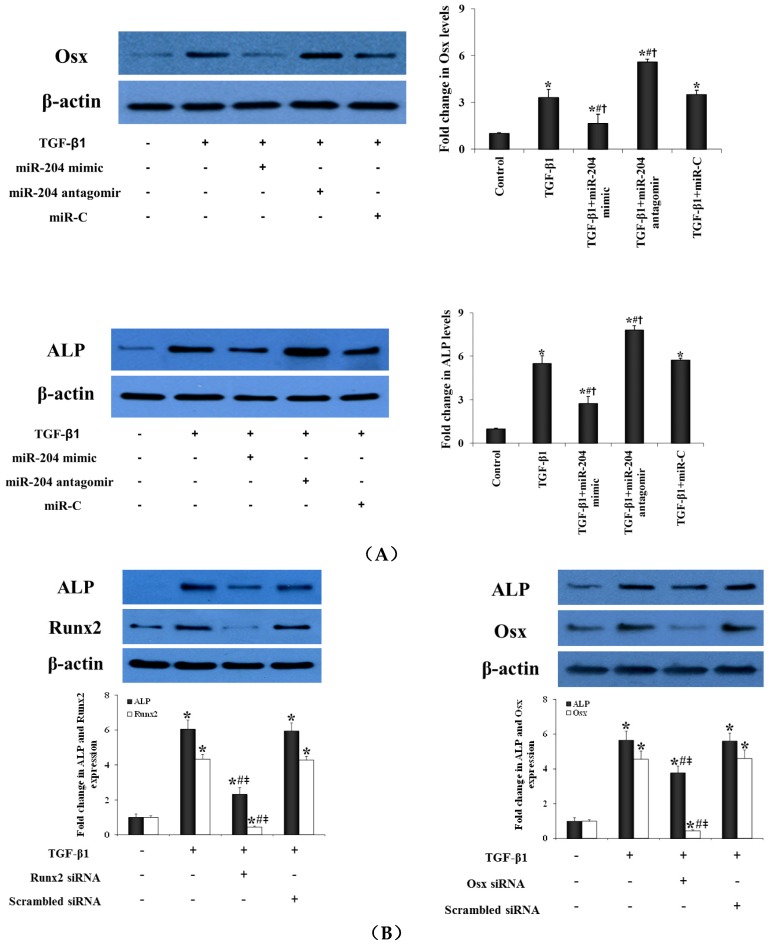
MiR-204 down-regulates the expression of both Runx2 and Osx expression in human AVICs to suppress pro-osteogenic reprogramming. (**A**) AVICs from normal aortic valves were treated with miR-204 mimic (5 nM), antagomir (50 nM) or irrelevant oligonucleotide (miR-C, 50 nM) for 24 h and then stimulated with TGF-β1 (0.005 μg/mL) for 24 to 48 h. Representative immunoblots and densitometric data show that miR-204 mimic suppressed, but miR-204 antagomir enhanced the expression of Runx2 and Osx at 24 h, as well as the levels of ALP at 48 h following treatment with TGF-β1. Mean ± SE; *n* = 6 distinct cell isolates; * *p* < 0.05 vs. untreated control; # *p* < 0.05 vs. TGF-β1 alone; ^†^ *p* < 0.05 vs. TGF-β1+miR-C. (**B**) AVICs from normal aortic valves were treated with specific siRNA (100 nM) for 48 h to knockdown Runx2 or Osx and then stimulated with TGF-β1 (0.005 μg/mL) for 48 h. Controls were pre-treated with scrambled siRNA (100 nM) and then stimulated with TGF-β1. Representative immunoblots and densitometric data show that knockdown of Runx2 markedly reduced the effects of TGF-β1 on ALP levels, whereas knockdown of Osx had a moderate effect. Mean ± SE; *n* = 6 distinct cell isolates; * *p* <0.05 vs. untreated control; # *p* < 0.05 vs. TGF-β1 alone; ≠ *p* < 0.05 vs. TGF-β1+scrambled siRNA.

We investigated whether miR-204 modulate AVIC pro-osteogenic transition induced by TGF-β1. We transfected normal AVICs with miR-204 mimic or antagomir and then exposed cells to TGF-β1. As shown in Figure 4A, miR-204 mimic suppressed the expression of Runx2, Osx, and ALP induced by TGF-β1. Conversely, miR-204 antagomir increased Runx2, Osx, and ALP levels in cells exposed to TGF-β1. These findings suggest that miR-204 inhibits TGF-β1-induced AVIC pro-osteogenic transition.

To determine the role of Runx2 and Osx in mediating the expression of the early osteoblastic biomarker ALP induced by TGF-β1, we applied siRNA specifically for human Runx2 or Osx before stimulation with TGF-β1. The results in Figure 4B show that knockdown of either Runx2 or Osx reduced ALP levels in cells exposed to TGF-β1. It is noteworthy that knockdown of Runx2 markedly reduced the effect of TGF-β1 on ALP expression, whereas knockdown of Osx had a moderate effect. Thus, both Runx2 and Osx play a role in mediating ALP expression induced by TGF-β1, and Runx2 occupies a major role.

### 2.5. Expression of MiR-204 Antagomir Promotes Calcium Deposit Formation in Aortic Valves

**Figure 5:**
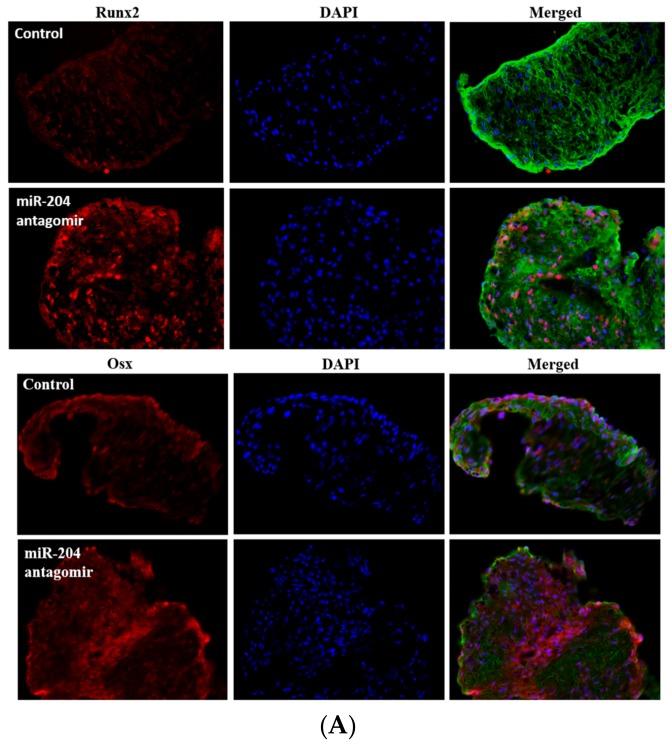

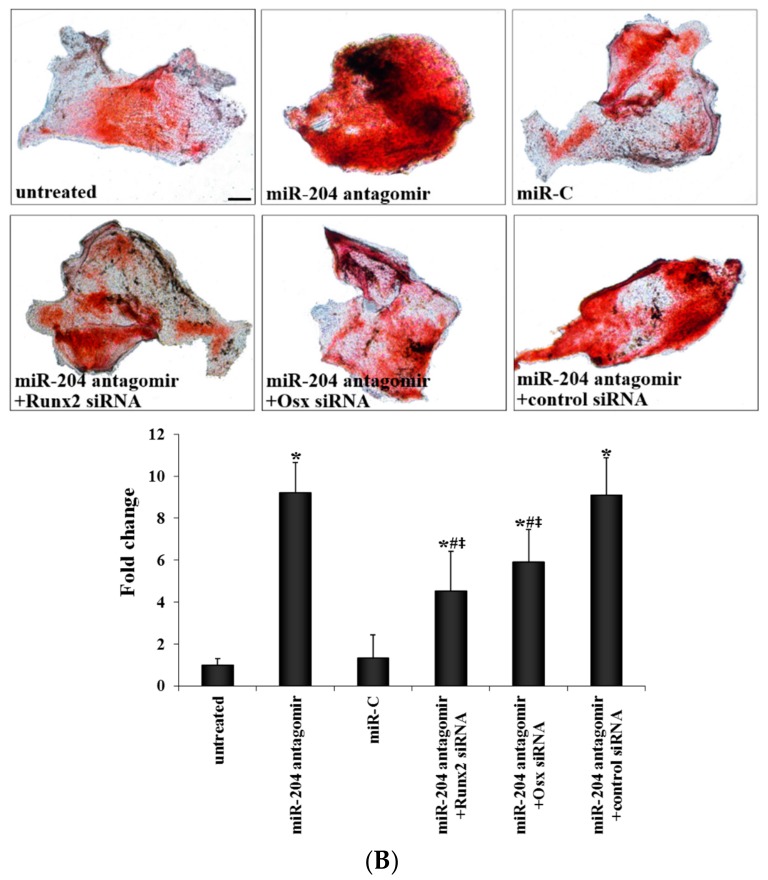
MiR-204 antagomir promotes aortic valve calcification through up-regulation of Runx2 and Osx expression. (**A**) Isolated mouse aortic valves were treated with irrelevant miR (control) or miR-204 antagomir for three days. Representative immunofluorescence images of 5 separate experiments show that miR-204 antagomir increases the levels of Runx2 and Osx. Runx2 and Osx are showing in red. The nuclei are showing in blue. Glycoproteins are showing in green to outline tissue and cells. Original magnification 40× objective. (**B**) Isolated mouse aortic valves were untreated or treated with irrelevant miR or miR-204 antagomir. Additional valves were treated with miR-204 antagomir in the presence or absence of siRNA (100 nM) specific to Runx2 or Osx. Then, all valves were incubated in the conditioning medium for 6 days. Representative images of Alizarin Red S staining and spectrophotometric analysis of Alizarin Red S stains show that miR-204 antagomir augmented the formation of calcium deposits (brick red and black) in mouse aortic valves. Knockdown of Runx2 or Osx attenuated calcium deposit formation caused by antagonizing miR-204. Mean ± SE; *n* = 5; * *p* < 0.05 vs. untreated (conditioning medium alone) or miR-C (irrelevant miR+conditioning medium); # *p* < 0.05 vs. miR-204 antagomir; **≠** *p* < 0.05 vs. miR-204 antagomir+control siRNA. Scale bar = 200 μm.

To determine whether inhibition of miR-204 promotes aortic valve osteogenic activity, we treated mouse aortic valve leaflets ex vivo by transfection of miR-204 antagomir and infection with lentivirus expressing miR-204 antagomir. As shown in Figure 5A, transfection of mouse aortic valves with miR-204 antagomir enhanced their expression of both Runx2 and Osx. Further, miR-204 antagomir augmented calcium deposition in mouse aortic valves cultured in the conditioning medium (Figure 5B). To further determine the role of Runx2 and Osx in valvular osteogenic activity, we treated mouse aortic valves with specific siRNA to knockdown Runx2 or Osx and examined calcium deposition. As shown in Figure 5B, knockdown of either Runx2 or Osx attenuated calcium deposition caused by miR-204 antagonization. The results suggest that inhibition of miR-204 up-regulates the expression of both Runx2 and Osx to promote valvular calcification.

## 3. Discussion

CAVD is a leading cardiovascular disease in the elderly. While the progression of CAVD to aortic stenosis is a chronic process and appears to be preventable, the mechanism underlying progressive aortic valve calcification remains unclear. In the present study, we identified miR-204 deficiency in human aortic valves affected by CAVD. The results of in vitro experiments demonstrate that miR-204 mimic suppresses the osteogenic activity in AVICs of diseased valves whereas miR-204 antagomir enhances the osteogenic activity in AVICs of normal valves. Moreover, miR-204 suppresses TGF-β1-induced ALP expression in human AVICs through inhibition of the expression of Runx2 and Osx. The results obtained from ex vivo experiments using mouse aortic valves revealed that antagonization of miR-204 promotes valvular calcification through up-regulation of the expression of Runx2 and Osx. These findings suggest that miR-204 is a negative regulator of valvular osteogenic activity and that miR-204 deficiency contributes to the mechanism of valvular calcification in CAVD. Further, miR-204 is capable of at least partly reversing the pro-osteogenic phenotype of AVICs of diseased valves and suppressing AVIC response to pro-osteogenic stimulation.

### 3.1. Reduced Expression of MiR-204 in Diseased Aortic Valves Contributes to the Mechanism Underlying AVIC Pro-Osteogenic Phenotype

AVICs are heterogeneous and display fibroblastic and myofibroblastic characters, and these cells are actively involved in aortic valve calcification and thereby play an important role in the mechanism of CAVD progression. MiRs regulate the expression of protein-coding genes and thus have critical roles in epigenetic regulation of gene expression and cell function. Previous studies reported altered expression of several miRs, including 26a, 30b, 146a, and 195, in diseased aortic valves and suggested that they may modulate AVIC osteogenic responses [26,27,28]. Since our systemic miR analysis of AVICs from diseased and normal valves shows markedly lower levels of miR-204 in AVICs of diseased valves [32], we compared miR-204 levels in normal human aortic valves and diseased human aortic valves in the present study. We observed that in normal human aortic valves miR-204 is localized primarily in the interstitial tissue and cells, and its levels are markedly lower in diseased human aortic valve tissue obtained from non-calcified areas. In addition, AVICs from the non-calcified areas of diseased valves also have much lower levels of miR-204, and miR-204 deficiency in AVICs of diseased valves correlates with their pro-osteogenic phenotype characterized by over-expression of ALP and exacerbated calcium deposit formation. It appears that miR-204 deficiency has a mechanistic role in mediating AVIC pro-osteogenic phenotype transition observed in diseased aortic valves.

ALP is a biomarker of early osteoblastogenesis. In osteoblast differentiation, ALP is expressed between the late osteoprogenitor and pre-osteoblast stages [33]. As ALP levels increase during early osteoblastogenesis [34], up-regulation of cellular levels of this protein is considered a biomarker of pro-osteogenic differentiation. In the present study, we found that expression of miR-204 mimic in AVICs of diseased valves increases miR-204 levels, reduces ALP to levels comparable to those of AVICs of normal valves and suppresses calcium deposit formation. Conversely, antagonization of miR-204 in AVICs from normal valves enhances the expression of Runx2, Osx, and ALP. Thus, two lines of evidence demonstrate that miR-204 is a negative regulator of osteogenic activity in human AVICs. Collectively, these findings suggest that miR-204 deficiency has a mechanistic role in elevation of AVIC osteogenic activity, and correction of miR-204 levels in AVICs of diseased valves is capable of reversing their pro-osteogenic phenotype.

### 3.2. MiR-204 Suppresses Valvular Osteogenic Activity through Down-Regulation of Runx2 and Osx

High levels of TGF-β1 have been observed in aortic valves affected by CAVD [20,35]. In addition, several studies demonstrate that TGF-β1 up-regulates the osteogenic activity in human AVICs [20,21,36,37]. Thus, TGF-β1 may play a role in the development and progression of aortic valve calcification associated with CAVD. We tested the hypothesis that miR-204 modulates the osteogenic response to TGF-β1 in human AVICs. An exposure to this pro-osteogenic factor up-regulated the expression of osteogenic transcription factors Runx2 and Osx, and increased the levels of osteogenic biomarker ALP. Interestingly, miR-204 mimic suppressed and miR-204 antagomir exacerbated the expression of Runx2, Osx, and ALP in human AVICs exposed to TGF-β1. Thus, modulation of cellular miR-204 levels or activity alters AVIC osteogenic response to TGF-β1. In review of the previous report that miR-204 suppresses AVIC osteogenic response to BMP-2 [29], it is reasonable to postulate that miR-204 suppresses AVIC osteogenic response to distinct pro-osteogenic stimuli through regulation of a key mediator of the response.

Runx2 is required for the expression of multiple osteogenic genes, including osteopontin, ALP, bone sialoprotein and osteocalcin [38] and miR-204 has been found to down-regulate Runx2 expression associated with mesenchymal progenitor cell differentiation to osteoblast [31]. In the present study, we observed that TGF-β1 up-regulates the expression of both Runx2 and Osx. We performed loss-of-function experiments to determine the relative role of Runx2 and Osx in mediating ALP expression induced by TGF-β1. Knockdown of Runx2 or Osx with specific siRNA reduced ALP levels following stimulation with TGF-β1. This observation is in agreement with previous reports that both Runx2 and Osx up-regulate the expression of the osteogenic biomarker ALP in mesenchymal stem cells and osteoblast precursor cells [39,40]. Our work demonstrates that miR-204 inhibits the expression of both Runx2 and Osx in human AVICs to suppress their osteogenic response to TGF-β1. It is noteworthy, however, that knockdown of Runx2 markedly reduced the effects of TGF-β1 on ALP expression, whereas knockdown of Osx had a moderate effect. It appears that Runx2 plays a major role in mediating AVIC osteogenic activity.

To further determine the role of miR-204 deficiency in elevating the osteogenic activity in aortic valves, as well as the mechanism that mediates the elevation of valvular osteogenic activity, we expressed miR-204 antagomirs in mouse aortic valves using lentivirus. While valves expressing control miR had a low level of mineralization activity as confirmed by calcium staining, treatment with lentiviral miR-204 antagomir resulted in a greater level of calcium deposition associated with elevated levels of Runx2 and Osx. Knockdown of either Runx2 or Osx attenuated calcium deposition in mouse aortic valves treated with lentiviral miR-204 antagomir. These findings provide further evidence that miR-204 is a negative regulator of osteogenic activity in the aortic valve and demonstrate that insufficient miR-204 function elevates valvular osteogenic activity through up-regulation of the expression of osteogenic transcription factors Runx2 and Osx. These findings also highlight the important role of miR-204 in regulating the expression of both Runx2 and Osx in the aortic valve.

### 3.3. Summary and Limitations

The present study demonstrates that greatly reduced levels of miR-204 in diseased human aortic valves contribute to the mechanism underlying AVIC pro-osteogenic phenotype and valvular osteogenic activity. Expression of miR-204 mimic in AVICs of diseased aortic valves reverses their pro-osteogenic phenotype. MiR-204 also suppresses TGF-β1-induced ALP expression in human AVICs through inhibition of the expression of both Runx2 and Osx, and inhibition of miR-204 up-regulates Runx2 and Osx to elevate valvular osteogenic activity. The results of this study suggest that miR-204 may have therapeutic potential for suppression of valvular calcification associated with CAVD progression.

One of the limitations of this study is its relatively small sample size (diseased aortic valves from eight patients with CAVD and normal aortic valves from eight explanted hearts of heart transplant recipients). To ensure the reproducibility of the observations, all in vitro experiments were repeated using cell isolates from at least six different donors. Another limitation is that we focused only on Runx2 and Osx, two well-known osteogenic transcription factors, although Sox4 and NFAT5 are indicated by bioinformatics analysis as potential targets of miR-204. Future studies are needed to identify other miR-204 targets that modulate the osteogenic activity in the aortic valve. In addition, recombinant TGF-β1 was applied as a pro-osteogenic stimulus in the in vitro experiments. As the concentration of TGF-β1 in diseased human aortic valves, particularly in the micro-environments surrounding AVICs, is currently unknown, we used a frequently cited concentration. However, it may be quite different from those in the diseased human aortic valves Sox4, Sox11, and NFAT5.

## 4. Materials and Methods

### 4.1. Materials

Antibodies against human and mouse Runx2 were purchased from Cell Signaling, Inc. (Beverly, MA, USA). Antibodies against both human and mouse Osx were purchased from Santa Cruz Biotechnology, Inc. (Santa Cruz, CA, USA). TGF-β1 and antibodies against ALP were purchased from R&D System (Minneapolis, MN, USA). Specific siRNAs for human Osx and Runx2 and lipofectamine 2000 were purchased from Life Technologies, Inc. (Grand Island, NY, USA). HiPerFect® transfection reagent, EndoFree Plasmid Maxi Kit, control miR, miR-204 mimics, and antagomirs were all obtained from Qiagen (Valencia, CA, USA). Lentivirus vector expressing miR-204 mimic or miR-204 antagomir, as well as Block-it lentiviral pol II miR RNAi expression plasmids were obtained from Invitrogen (Grand Island, NY, USA). TransDux transduction reagent was obtained from System Biosciences (Mountain View, CA, USA). Medium 199 and cell culture supplements were purchased from Lonza (Walkersville, MD, USA). All other chemicals and reagents were purchased from Sigma-Aldrich Chemical Co. (St Louis, MO, USA).

### 4.2. Isolation, Culture and Treatment of Human AVICs

Calcified aortic valves were collected during aortic valve replacement surgeries from eight patients with CAVD (seven males and one female, age 58.4 ± 11.6 years). All of these diseased valves are tricuspid. Normal aortic valves were from explanted hearts of eight patients (eight males, age 50.3 ± 16.1 years) undergoing heart transplantation due to cardiomyopathy. The valve leaflets from the explanted hearts of transplant recipients were thin and did not have histological abnormality. This study was approved by the University of Colorado Multiple Institution Review Board (IRB Protocol 08-0280; approval Date: 1/05/2016). All subjects gave their informed consent for the use of their aortic valves for this study. The investigations were carried out following the rule of the Declaration of Helsinki of 1975, revised in 2013.

AVICs were isolated and cultured using a previously described method [37,41,42]. Briefly, valve leaflets were subjected to sequential digestions with collagenase. A high concentration of collagenase (2.5 mg/mL) was used to remove endothelial cells. Then, the tissue was digested in a solution containing 0.8 mg/mL of collagenase to free the interstitial cells, and AVICs in the solution were harvested by centrifugation. Immunofluorescence staining for von Willebrand factor confirmed that AVIC isolates obtained following this protocol have no endothelial cell contamination [41]. Cells were maintained in M199 growth medium supplemented with 10% fetal bovine serum, penicillin G (100 units/mL), streptomycin (100 mg/mL) and amphotericin B (0.25 µg/mL). Cells of passage 3 to 6 were used for the experiments when the cultures reached 80% to 90% confluence.

To determine the role of miR-204 in regulating AVIC expression of Runx2, Osx, and ALP, cells were transfected with miR-204 mimic (5 nM), miR-204 antagomir (50 nM), or control miR (50 nM). 

To determine the role of miR-204 in modulating the effect of TGF-β1 on AVIC expression of Runx2, Osx, and ALP, cells were transfected with miR-204 mimic (5 nM), miR-204 antagomir (50 nM), or control microRNA (50 nM) with or without a stimulation with TGF-β1.

To determine the role of Runx2 and Osx in mediating the effect of TGF-β1 on AVIC expression of ALP, cells were pretreated with Runx2 siRNA or Osx siRNA, and then stimulated with TGF-β1.

### 4.3. Mouse Aortic Valve Culture

Male C57BL/six mice (4 to 6 months) were obtained from the Jackson Laboratory (Bar Harbor, ME, USA), The use of mice for collection of aortic valves was approved by the Institutional Animal Care and Use Committee of the University of Colorado Denver (Protocol B-40516-08-2D; approval date: 24/08/2016), and the care and use of animal for this investigation conform to the Guide for the Care and Use of Laboratory Animals (National Research Council, revised 1996).

Thoracotomy was performed under anesthesia by intraperitoneal injection of ketamine (60–80 mg/kg) and xylazine (12 mg/kg). Beating hearts were removed and aortic valves were collected from the extracted hearts under a dissecting microscope. The leaflets obtained were comparable in size. Ex vivo culture of mouse aortic valve leaflet was performed following the method described by Peacock and colleagues. [43]. In brief, each valve leaflet was placed on a piece of filter membrane (0.2 µm pore size) and immersed in M199 growth medium supplemented with 10% fetal bovine serum, penicillin G (100 units/mL), streptomycin (100 mg/mL), and amphotericin B (0.25 µg/mL). Conditioning medium (growth medium supplemented with 10 mmol/L β-glycerophosphate, 10 nmol/L vitamin D_3_, 10 nmol/L dexamethasone, and 8 mmol/L CaCl_2_) was applied to promote calcium deposition. Treatment with lentiviral miR-204 antagomir or irrelevant miR was applied to determine the effect of inhibition of miR-204 function on aortic valve calcification.

### 4.4. Preparation of Recombinant Lentiviral Supernatants and Lentiviral Transduction

The procedures were performed as described in our previous study [32]. Briefly, Block-it lentiviral pol II miR RNAi expression plasmids were amplified using standard bacterial transformation and purified using EndoFree Plasmid Maxi Kit. Lentivirus expressing human and mouse miR-204 mimic or antagomir was generated by Lipofectamine 2000 co-transfection of 293T-cells. After 48 h, lentiviral supernatants were collected and concentrated.

Lentiviral expression of miR-204 mimic or antagomir was applied to determine their effect on calcium deposition. Human AVICs and mouse aortic valves were infected with lentivirus expressing miR-204 mimic or antagomir, and then cultured in conditioning medium for 14 and six days, respectively. Calcium deposition was evaluated using Alizarin red S staining.

### 4.5. Immunoblotting

Immunoblotting was performed, as described previously [44], to analyze Runx2, Osx, and ALP. Cells were lysed in a sample buffer (100 mmol/L Tris-HCl, pH 6.8, 2% SDS, 0.02% bromophenol blue and 10% glycerol). Proteins in cell lysate were fractioned by 4–20% SDS-PAGE and transferred to PVDF membranes. Following incubation in a 5% skim milk solution, membranes were treated with primary antibodies and subsequently with peroxidase-linked secondary antibodies. Protein bands were developed using the enhanced chemiluminescence system. β-actin levels were examined for normalization of loading. Band density was analyzed using the Image J software (Wayne Rasband, National Institutes of Health, Bethesda, MD, USA).

### 4.6. Real-Time RT-PCR Analysis

Trizol reagent and a Qiagen miRNeasy Mini Kit (Valencia, CA, USA) were used to isolate total RNA. Reverse transcription (RT) and PCR analysis were performed using iScriptTM cDNA Synthesis Kit (Bio-Rad, Hercules, CA, USA), Qiagen miScript II RT Kit, iQ™ SYBR^®^ Green Supermix and Qiagen miScript SYBR^®^ Green PCR Kit according to the manufacturers’ instructions. The following primers were used to amplify specific cDNA fragments: miR-204 (forward: 5′-CCCCTTCCCTTTGTCATCCTATGCCT-3′; reverse: miScript Universal Primer); U6 (forward: 5′-CTCGCTTCGGCAGCACA-3′; reverse: miScript Universal Primer). MiR-204 levels were quantified by real-time PCR using the iQ™ 5 Multicolor Real-time PCR Detection system (Bio-Rad). MiR-204 levels normalized to U6 were calculated using the 2^−ΔΔCT^ method [45].

### 4.7. In Situ Hybridization

Paraffin sections of normal and calcified human aortic valve tissue were mounted on glass slides and deparaffinized. Sections were treated with proteinase-K (10 μg/mL) at 37 °C for 5 min, pre-hybridized in hybridization solution (formamide 50%, 5×SSC, Tween 20 0.1%, heparin 50 µg/mL and Yeast tRNA 500 µg/mL) at 53 °C for 60 min, and then hybridized with 40 nM miR-204 probe and U6 probe. Following stringent washes with 5×, 1× and 0.2× SSC buffers at 53 °C for 30 min, sections were incubated in digoxigenin blocking reagent in blocking buffer containing 2% sheep serum and 2 mg/mL bovine serum albumin at room temperature for 60 min. Then, sections were incubated with alkaline phosphatase-conjugated anti-digoxigenin (diluted 1:2000 in blocking reagent from Roche, Indianapolis, IN, USA) at room temperature for 60 min. Enzymatic development was carried out using Benjamin Moore Purple substrate (Roche) at room temperature for 72 h.

### 4.8. Histology and Immunofluorescent Staining

Cryosections of mouse valve tissues and paraffin sections of human aortic valve tissues were stained with hematoxylin and eosin (H–E) and visualized in a bright field using a Nikon microscope (Tokyo, Japan).

Immunofluorescence staining was performed, as described previously [46], to detect Runx2 and Osx in mouse aortic valves. After permeabilization with an ethanol/acetone mixture, tissue sections were fixed in 4% paraformaldehyde, incubated with a rabbit polyclonal antibody against mouse Runx2 or Osx overnight at 4 °C. After washing with phosphate-buffered saline (PBS), cells were treated with Cy3-tagged secondary antibody. Nuclei were counterstained with bisbenzimide (4′,6-diamidino-2-phenylindole, DAPI), and glycoproteins were stained with Alexa 488-tagged wheat germ agglutinin (WGA). Microscopy was performed using a Leica DM 5500 microscope (Leica Microscopy und System GmbH, Wechsler, Germany).

### 4.9. Staining for Calcium Deposits

Alizarin red S staining was performed using the method described previously [37,47]. Briefly, cells were washed with PBS and fixed in 4% paraformaldehyde for 15 min, followed by incubation with 0.2% Alizarin red solution (pH 4.2) at room temperature. Following washes with distilled water, Alizarin red stains were examined and photographed with a Nikon Eclipse TS100 microscope (Tokyo, Japan). For quantitative analysis, the red stains were bleached with 10% acetic acid and assessed spectrophotometrically at 450 nm [48].

### 4.10. Statistical Analysis

All results are expressed as mean ± SE. Group comparisons were done using SPSS 13.0 software with one-way analysis of variance (ANOVA) and the post hoc Bonferroni/Dunn test. Differences were confirmed using the Kruskal-Wallis H test. A difference was considered significant at *p* ≤ 0.05.
